# Papillary meningioma with prominent flow voids

**DOI:** 10.4102/sajr.v28i1.2778

**Published:** 2024-03-22

**Authors:** Mousam Panigrahi, Narendra K. Bodhey, Vandita Y. Singh, Lokesh Nehete

**Affiliations:** 1 Department of Radiodiagnosis, All India Institute of Medical Sciences, Raipur, India; 2 Department of Pathology and Laboratory Medicine, All India Institute of Medical Sciences, Raipur, India; 3 Department of Neurosurgery, All India Institute of Medical Sciences, Raipur, India

**Keywords:** papillary meningioma, meningioma, flow voids, magnetic resonance perfusion, prognosis

## Abstract

This report presents an extremely rare case of papillary meningioma with prominent flow voids and increased perfusion parameters on MRI in a 28-year-old male presenting with headache. This knowledge helped the neurosurgeon to minimise intra-operative blood loss and achieve a favourable post-surgical outcome.

**Contribution:**

A rare case of papillary meningioma and its differentiating features from typical meningiomas have been discussed considering its implications for management as well as prognostication to reduce morbidity and mortality.

## Introduction

Papillary meningioma (PM) is a rare subtype of World Health Organization (WHO) grade III meningioma with a highly aggressive clinical course, local recurrence and cerebrospinal fluid (CSF) metastasis, and a poor prognosis.^1^ It represents less than 1% of all meningiomas.^1^ The presence of flow voids in an extra-axial tumour favours the diagnosis of hemangiopericytoma.^2^ However, we report this extremely rare case of PM with prominent flow voids and increased perfusion parameters on MRI. Presently, only two reports were found in the literature about PM associated with flow voids.^3,4^ The different MRI characteristics of PMs have also been discussed because MRI studies on PMs are very limited due to the relative rarity of the lesion.

## Case presentation

A 28-year-old male presented with persistent dull aching left parietal headache for a period of a year. General physical examination and systemic examination were unremarkable. No focal neurological deficit was evident.

**FIGURE 1:**
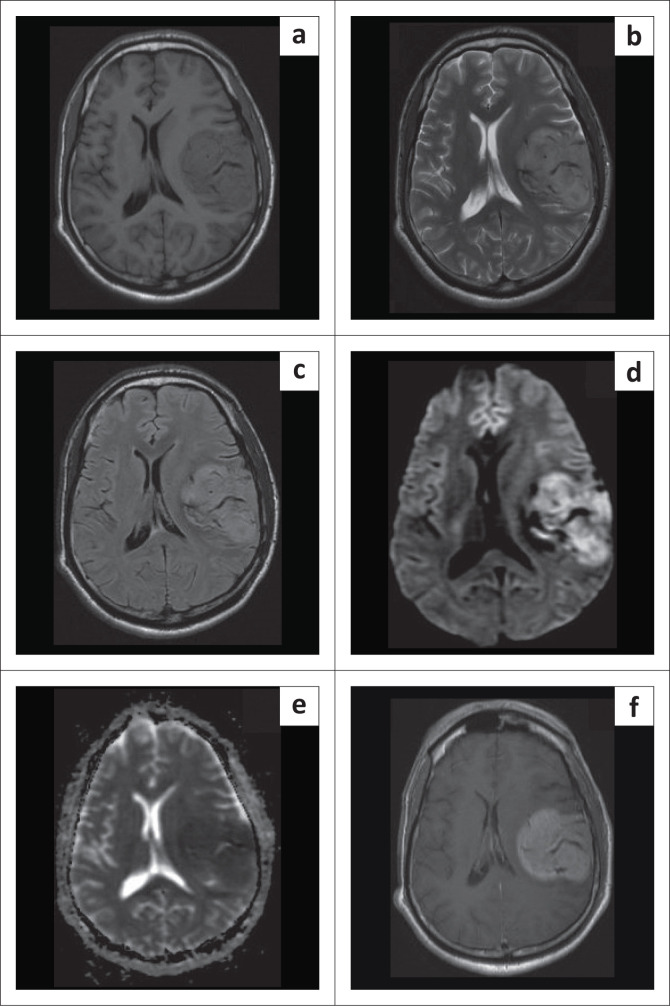
MRI brain shows a well-defined, lobulated, extra-axial lesion along left parietotemporal region. It appears isointense to grey matter on T1W (a) and T2W (b), and slightly hyperintense on fluid-attenuated inversion recovery (FLAIR) images (c). Multiple dilated vascular channels (flow voids) were seen within the lesion. It shows diffusion restriction on diffusion weighted imaging (DWI) (d) with low apparent diffusion coefficient (ADC) (e) values. It shows fairly homogeneous post-contrast enhancement (f).

**FIGURE 2:**
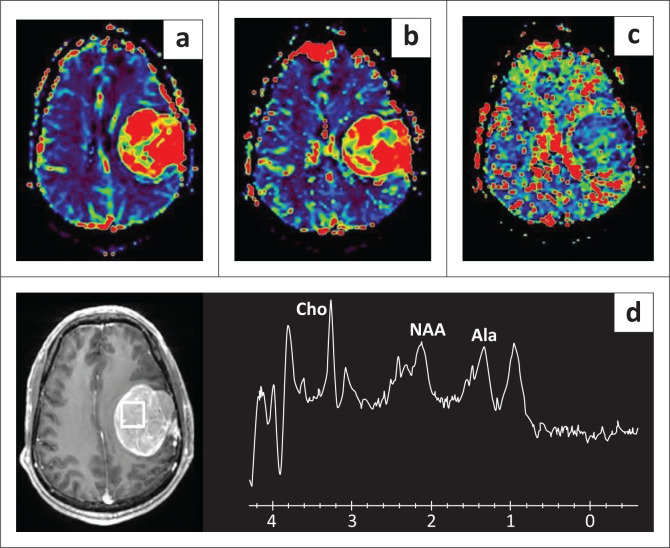
Magnetic resonance (MR) perfusion shows high relative cerebral blood flow (rCBF) (a) and relative cerebral blood volume (rCBV) (b) with reduced MTT (c). MRS (d) shows elevated choline and alanine with reduced NAA.

The MRI brain revealed a well-defined, lobulated, extra-axial lesion measuring 5.5 cm × 5.5 cm × 6.1 cm along the left parietotemporal dura. Multiple dilated vascular channels (flow voids) were seen within the lesion. Prominent branches could be traced to the left middle meningeal artery and the M3 segment of the left middle cerebral artery (MCA). There were no intratumoural or peritumoural cysts and no calcification or peritumoural oedema. Diffusion weighted imaging (DWI) showed restricted diffusion with a low apparent diffusion coefficient (ADC) of 0.468 × 10^−3^ mm^2^/s (Figure 1). The lesion showed increased perfusion relative to normal parenchyma (rCBF: 65.21; rCBV: 9.67; mean transit time [MTT]: 8.93 s). Arterial spin labelling (ASL) perfusion revealed cerebral blood flow (CBF) of 292.4 mL/100 g/min. Magnetic resonance spectroscopy showed elevated choline and alanine peaks (Figure 2). Based on these findings, a diagnosis of meningioma was made, with a suspicion of an atypical or malignant variant because of the low ADC and increased perfusion. However, the possibility of hemangiopericytoma was also considered because of the prominent flow voids.

Following the preoperative investigations, the tumour was excised through a left parietotemporal craniotomy approach. It was a highly vascular extra-axial mass (attached to the dura), which was soft to firm in consistency and reddish in appearance. The surgery was uneventful. No significant blood loss occurred as the feeding vessels were identified and isolated first, based on the MRI findings.

**FIGURE 3:**
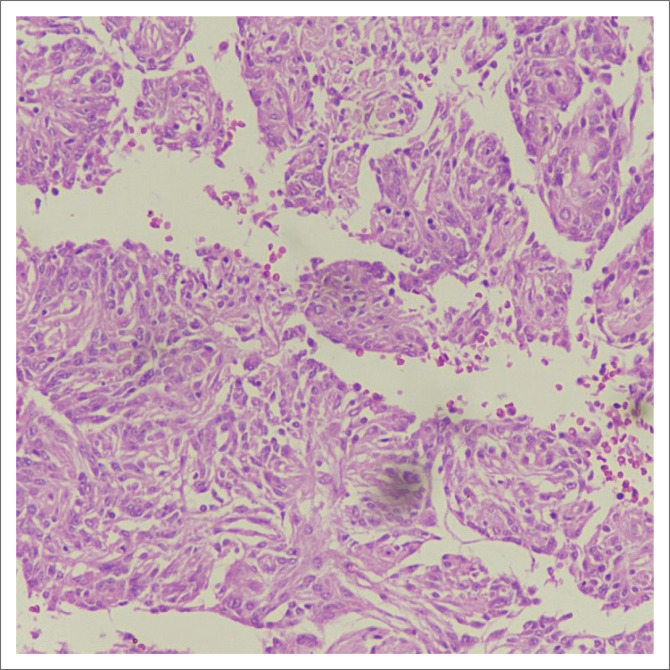
The histopathological photomicrograph shows sheets of cells arranged in a predominantly papillary pattern with perivascular pseudorosettes. Tumour cells show moderate eosinophilic cytoplasm and vesicular nuclei. Atypical mitoses of upto 16/10 high power field (HPF) were noted. There were foci of small cell formation and incipient necrosis.

Subsequent histopathological examination (Figure 3) revealed sheets of cells arranged in a predominantly papillary pattern with perivascular pseudorosettes. The tumour cells showed moderate eosinophilic cytoplasm and vesicular nuclei. Atypical mitoses of up to 16/10 high power field (HPF) were noticed. There were foci of small cell formation and incipient necrosis. Immunohistochemistry of the tumour cells was diffusely positive for CD34 and epithelial membrane antigen (EMA) and negative for glial fibrillary acidic protein (GFAP). Ki67 labelling index was 10% – 12%. These findings established the diagnosis of PM, WHO grade III.

## Discussion

Papillary meningioma is a rare malignant meningioma subtype with a predilection for young male patients, similar to this case.^3^ Papillary meningiomas present with variable clinical symptoms including seizures, headache, blurred vision, dizziness, tinnitus, vomiting and hemiparesis depending on the location of the tumour and surrounding oedema.^4^ In this case, the mass was located along left parietotemporal convexity and headache was the only presenting symptom. Papillary meningiomas are mostly located along the cerebral convexities.^5,6^ Unusual sites of PMs include the brainstem, foramen magnum, oculomotor nerve, jugular foramen and posterior fossa.^7,8^

Yu et al. reported an irregular or lobulated shape, similar to this case, which is ascribed to the inhomogeneous distribution of multiplying cells in the PMs and leads to an imbalanced cell density and an uneven intratumoural pressure.^4^ Previous studies reported an average maximum diameter of around 4.6 cm – 5.0 cm (range: 2 cm – 8 cm).^4,5^ This case had a maximum diameter of 6.1 cm.

Most of the PMs show marked heterogeneous post-contrast enhancement.^4,5^ However, this case showed fairly homogeneous enhancement except for the prominent vascular channels. These vascular channels corresponded to flow voids on T2-Weighted images. Flow voids in PM represent fast blood flow through either dilated veins or dilated arteries.^3^ Papillary meningiomas can secrete many proangiogenic factors, such as vascular endothelial growth factor (VEGF) leading to more vessel proliferation.^3^ High vascularity of PM may lead to significant haemorrhage during surgery. One previously reported case resulted in severe intra-operative bleeding and death of the patient on the second day after surgery.^3^ In the presented case, the feeding vessels were identified at the time of the MRI and secured intraoperatively, resulting in no significant bleeding and a favourable surgical outcome. Pre-operative embolisation of the feeding vessels may also be considered in such cases. Vascular neoplasms (like hemangioblastoma, hemangiopericytoma), high-flow vascular lesions (like arteriovenous malformation and aneurysms), benign neoplasms (like Schwannoma and pituitary adenoma) and malignant neoplasms (like glioblastoma) can also demonstrate flow voids.^3^ Of these, the closest differential for PM is haemangiopericytoma, an extra-axial, multilobulated heterogeneously enhancing lesion. Unlike meningiomas, these tumours may show signal voids, bone erosion and a narrow-based attachment.^2^ Although flow voids are not specific for PMs, they can help in differentiating PMs from typical meningiomas.^4^

Yu et al. reported an unclear tumour-brain interface (TBI) in seven of nine lesions.^4^ Cerebrospinal fluid space and/or blood vessels around the lesion appear as a low-intensity border on T1-Weighted images representing the TBI.^9^ Unclear TBI in PMs indicates the lack of these physiological barriers between the tumour and adjacent brain parenchyma and proposes tight adhesion or tumour invasion of the brain.^10^ However, in this case, the TBI was clear with well-defined tumour borders, indicating a non-infiltrating nature.

Intratumoural or peritumoural cystic change is rarely seen in typical meningiomas but has been frequently reported in PM.^4,11^ Peritumoural cysts may occur because of widening of the subarachnoid space, the final stage of intense peritumoural oedema, fibroblastic proliferation, reactive gliosis, or disturbance of CSF resorption. Intratumoural cysts result from haemorrhage within the tumour, ischaemic necrosis or microcystic degeneration.^12^ In this case, no cystic changes or haemorrhages were seen.

Peritumoural oedema is commonly associated with PMs.^4,5^ Tamiya et al. reported that cortical infiltration, defined by the loss of the arachnoid membrane on MRI, together with arterial supply from the pial vessels on angiography, is significantly associated with peritumoural brain oedema in primary intracranial meningiomas.^13^ Indistinct TBIs and irregular tumour shapes indicate interruptions of the arachnoid layer. In this case, the TBI was clear and hence there was no peritumoural oedema.

Papillary meningiomas are frequently associated with osseous changes, including bone hyperostosis or destruction.^5^ No such osseous changes were seen in this case.

Not many studies have been carried out on advanced MR imaging in meningiomas. A previous attempt by Yang et al. to evaluate the grade of meningiomas by MR perfusion with the measurement of relative cerebral blood volume (rCBV) of the tumour parenchyma was not successful.^14^ Another study demonstrated that the mean maximal rCBV and corresponding relative mean time to enhance (rMTE) values of peritumoural oedema of atypical meningiomas were greater than those of typical meningiomas with statistically significant differences between the two groups (*p* < 0.05).^15^ This might be ascribed to angiogenesis and tumour invasion in the adjacent brain parenchyma. Another recent study demonstrated higher CBF in atypical meningiomas on ASL perfusion (*p* < 0.05) with a cut off of 276.75 mL/100 g/min.^16^ In the presented case, the tumour showed higher CBF (ASL), rCBV and relative cerebral blood flow (rCBF) with lower MTT than the brain parenchyma suggesting the possibility of an atypical nature of the lesion.

Previous studies with DWI demonstrated lower ADC values in malignant meningiomas compared with benign meningiomas (except for densely calcified meningiomas) or normal brain parenchyma.^17^ In the current case as well, the tumour showed a lower ADC value when compared to normal white matter, which was less than the cut off proposed by Nagar et al. (0.8 × 10^−3^ mm^2^/s), raising the suspicion of an atypical meningioma.^17^

On MR spectroscopy, meningiomas show elevated Cho and decreased N-acetyl aspartate (NAA). Prominent alanine is considered a spectroscopic signature for meningiomas.^18^ The MRS in this case, was consistent with these findings, providing a pre-operative confirmation of the lesion being meningioma.

Histological hallmarks of the PMs consist of pleomorphism, foci of necrosis, atypical mitosis and a perivascular pseudorosette pattern.^19^ Li et al. demonstrated EMA, vimentin and CD34 positivity in all cases of PMs.^5^ The findings of the current study were consistent with previous reports. No brain invasion was seen in this case suggested by GFAP negativity and the clear TBI.

The post-operative outcome of PMs is poor because of brain parenchymal invasion, local recurrences and metastasis (in the liver and lungs).^19^ Also, severe intra-operative bleeding can lead to poor outcomes in highly vascularised PMs.^3^

This case represents one of the very few reported cases of PM with prominent flow voids. High vascularity of the lesion is also demonstrated by increased MR perfusion parameters (high rCBF and rCBV, low MTT). Although this case did not have the classic features of a grade III meningioma on conventional MR imaging, a lobulated extra-axial lesion with prominent flow voids, increased perfusion and low ADC in a young male patient can raise the suspicion of malignancy.

## Conclusion

Papillary meningioma is a rare malignant variant with implications for management as well as prognostication to reduce morbidity and mortality. The presence of prominent flow voids, increased perfusion and low ADC in a lobulated extra-axial lesion in a young male patient should raise the suspicion of this variant even in the absence of the classic features of a grade III meningioma on conventional MR imaging. Considering the relative rarity of PM, multicentric studies may be conducted to study this lesion in detail, including advanced MRI features.
